# Laparoscopic appendectomy versus open appendectomy for suspected appendicitis during pregnancy: a systematic review and updated meta-analysis

**DOI:** 10.1186/s12893-019-0505-9

**Published:** 2019-04-25

**Authors:** Seung Hwan Lee, Jin Young Lee, Yoon Young Choi, Jae Gil Lee

**Affiliations:** 1 Department of Surgery, Yonsei University College of Medicine, 50-1 Yonsei-ro, Seodaemun-gu, Seoul, 03722 Republic of Korea; 2 Department of Trauma Surgery, Trauma Center, Chungbuk National University Hospital, Cheongju, Republic of Korea

**Keywords:** Pregnancy, Appendicitis, Laparoscopy, Appendectomy

## Abstract

**Background:**

Recently, laparoscopic appendectomies (LAs) have been widely performed instead of open appendectomies (OAs) during pregnancy. However, concerns about the safety of LA during pregnancy remain. This systematic review and meta-analysis aimed to evaluate the current evidence relating to the safety of LA versus OA for suspected appendicitis during pregnancy.

**Methods:**

Comprehensive literature searches were conducted using the PubMed, EMBASE, and Cochrane Library databases to identify articles describing LA versus OA in pregnancy, without restrictions regarding the publication date. The primary endpoints were fetal loss and preterm delivery.

**Results:**

After screening 801 studies, 22 comparative cohort studies were included in the analysis, which involved 4694 women, of whom 905 underwent LAs and 3789 underwent OAs. Fetal loss was significantly higher among those who underwent LAs compared with those who underwent OAs, and the pooled odds ratio (OR) was 1.72 (95% confidence interval [CI]: 1.22–2.42) without heterogeneity. The sensitivity analysis showed that the effect size was influenced by one of the studies, because its removal resulted in there being no significant difference between LA and OA with respect to the risk of fetal loss (OR 1.163, 95% CI: 0.68–1.99; *P* = 0.581). A significant difference was not evident between LA and OA with respect to preterm delivery (OR 0.76, 95% CI: 0.51–1.15), a result that did not change following the sensitivity analysis. The patients who underwent LA had shorter hospital stays (mean difference − 1.01, 95% CI: -1.61–-0.41) and a lower wound infection risk (OR 0.40, 95% CI: 0.21–0.76) compared with those who underwent OA.

**Conclusion:**

It is not reasonable to conclude that LA in pregnant women might be associated with a greater risk of fetal loss. The difference between LA and OA with respect to preterm delivery was not significant.

## Background

Acute appendicitis is the most common nonobstetric surgical problem that occurs during pregnancy, and its incidence varies widely, with rates ranging from 1.8 to 41 per 10,000 pregnancies [1–6]. The incidence of appendicitis has been reported to be higher during the second trimester than during the first or third trimesters of pregnancy [2, 4, 7–10]. Diagnosing acute appendicitis during pregnancy is challenging for surgeons, because of difficulties associated with nonspecific abdominal symptoms, and the physiologic leukocytosis and the anatomic changes in the appendix that occur during pregnancy.

Appendicitis during pregnancy has been reported to be associated with poor pregnancy outcomes, including fetal loss, preterm delivery, and perinatal morbidity and mortality [11]. Fetal loss occurs in 20% of women with complicated appendicitis compared with 1.5% of women with uncomplicated appendicitis [12–15]. The preterm delivery rate has been reported to be between 7.5 and 30.0%, and preterm delivery occurs more frequently in women with perforated appendicitis [16–26].

Open appendectomy (OA) has been performed on patients with acute appendicitis of both sexes and of all ages, including pregnant women. Moreover, laparoscopic appendectomy (LA) has also become a standard procedure for acute appendicitis since it was first performed in 1983 [27]. Although pregnancy was considered an absolute or relative contraindication for laparoscopic procedures initially, LA has recently been routinely performed in pregnant women in accordance with the recommendations in the guidelines published by the Society of American Gastrointestinal and Endoscopic Surgeons (SAGES) [28]. However, a systematic review [5] and a meta-analysis [4] concluded that there was low grade evidence to suggest that LA in pregnant women might be associated with a greater risk of fetal loss. Therefore, the optimal surgical approach for acute appendicitis during pregnancy remains a matter of debate. Thus, the aim of this systematic review and updated meta-analysis was to evaluate the current evidence regarding the safety of LA versus OA for suspected appendicitis during pregnancy.

## Methods

This systematic review was conducted and is reported according to the Preferred Reporting Items for Systematic Reviews and Meta-Analyses guidelines [29].

### Search strategy and study selection

Three major electronic medical databases, namely, PubMed, the Cochrane Central Register of Controlled Trials, and EMBASE, were comprehensively searched to find suitable studies using the following search terms: “pregnancy”, “pregnant women”, “appendicitis”, “appendectomy”, and “laparoscopy”. We restricted the searches to studies that were conducted on human subjects and those that were published in English, but there was no restriction regarding the publication date.

The present review included any comparative studies that compared the outcomes from LA and OA for appendicitis in pregnant patients. Those studies with at least one pregnancy outcome, for example, fetal loss, preterm delivery, birth weight, or the Apgar score, or one surgical outcome, for example, the wound infection rate, the intra-abdominal abscess rate, the operative time, or the length of stay (LOS), were included. Case series, review articles, and articles written in languages other than English were excluded from this review. When there were duplicate publications, the study with the largest number of subjects was selected.

The titles and the abstracts of the extracted studies were reviewed independently by two researchers (SHL and JYL). The complete manuscripts were reviewed if the abstracts did not provide enough information to indicate suitability for the study. The studies were finally included in the systematic review and meta-analysis after full-text evaluations were performed independently. Any discrepancies were resolved through discussion.

### Study outcomes and data extraction

This study’s primary endpoints were the pregnancy outcomes, including fetal loss and preterm delivery. The data extracted that included the publications’ general data (author, year of publication, and journal), the studies’ characteristics (the study’s design, the study period, and the sample size), the baseline characteristics of the studies’ populations (age, gestational age at surgery, and delivery type), the pregnancy outcomes (fetal loss, preterm delivery, birth weight, and the Apgar score), and the surgical outcomes (wound infection and intra-abdominal abscess rates, operative time, LOS, and the presence of complicated appendicitis), were summarized and analyzed. All of the data were cross-checked independently by two authors (SHL and JYL).

### Statistical analysis

The statistical analyses were performed using Review Manager, version 5.3 (RevMan; Nordic Cochrane Centre, Cochrane Collaboration, Copenhagen, Denmark) and Comprehensive Meta-Analysis, version 2 (Biostat, Englewood, NJ, USA). For the continuous outcomes, the results were pooled using the inverse-variance method and the mean differences (MDs) and their 95% confidence intervals (CIs) were calculated. For the dichotomous outcomes, the pooled odds ratios (ORs) and their 95% CIs were calculated using the Mantel-Haenszel method. The heterogeneity of the effect size across the studies was tested using Cochran’s Q test, with the significance level set at *P* < 0.10, and an I^2^ statistic with a value of ≥50% was considered to indicate substantial heterogeneity. A fixed effects model was used to pool the results when heterogeneity was not suspected, otherwise, a random-effects model was used. Publication bias was evaluated by assessing funnel plot symmetry. A sensitivity analysis, which involved repeating the sequential pooling outcomes while excluding each study in turn, was performed to evaluate whether the overall results were robust in relation to the excluded studies. Meta-regression analyses were performed to examine whether the primary outcomes were associated with other characteristics of the studies, for example, the publication year, complicated appendicitis, the gestational age, the pregnancy trimester, or a negative appendectomy.

## Results

### Search results

**Fig. 1:**
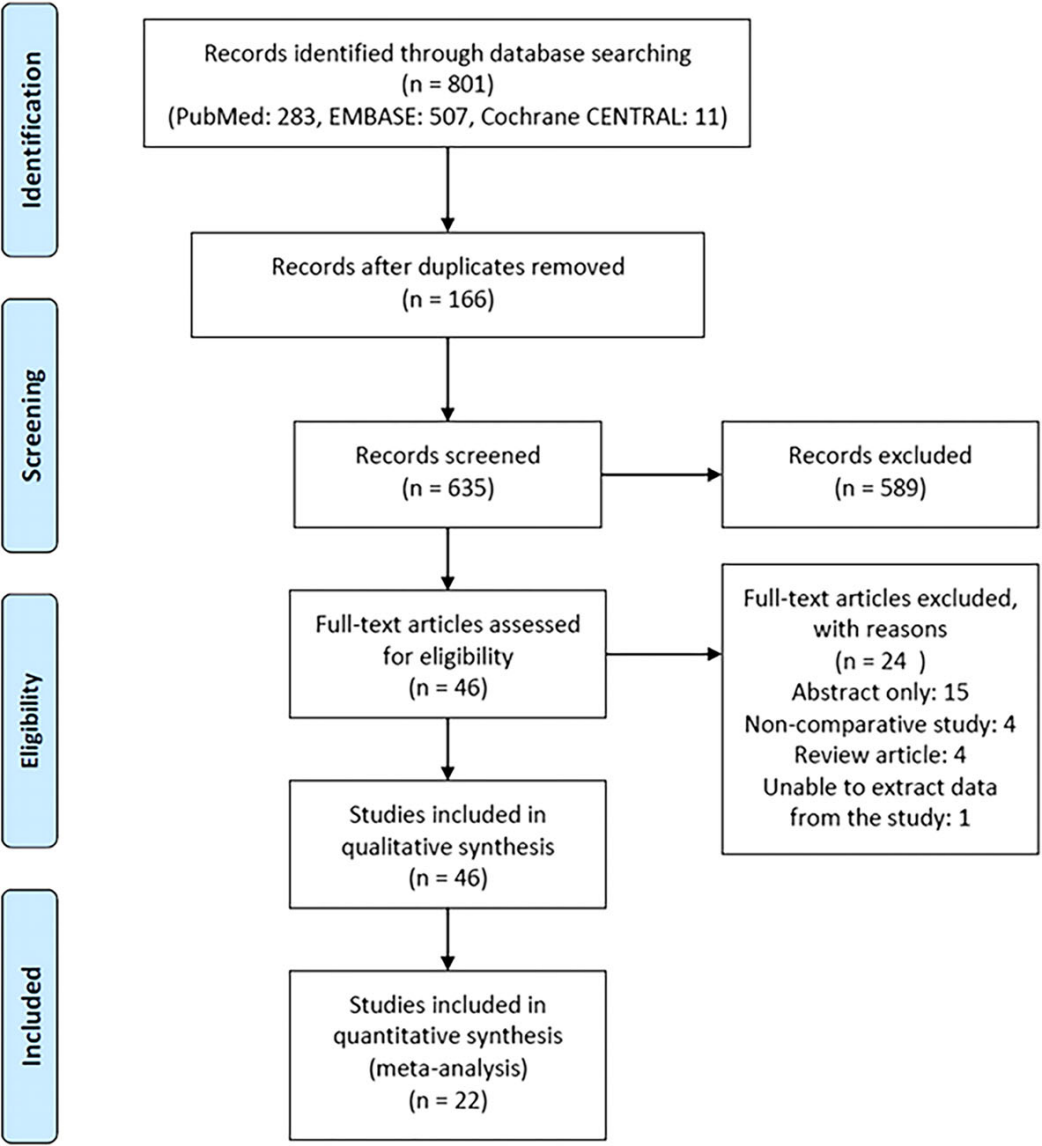
Flow diagram of the literature search and study selection

The study selection flow diagram is shown in Fig. 1. In total, 801 studies were identified. After screening the titles and the abstracts, 46 articles underwent full-text assessments, which led to the exclusion of 24 studies; therefore 22 comparative studies involving a total of 4694 women, of whom 905 underwent LAs and 3789 underwent OAs, were eligible for inclusion.

### Study characteristics

The characteristics of the included studies are presented in Table 1 [3, 16–26, 30–39]. Three studies [33, 36, 38] were comparative prospective cohort studies and nineteen studies [3, 16–26, 30–32, 34, 35, 37, 39] were comparative retrospective reviews of patients’ medical records. The studies were conducted between 1996 and 2016 in the United States of America (*n* = 9) [25, 26, 30, 33–35, 37–39], Korea (*n* = 4) [16, 20, 22, 23], Israel (*n* = 3) [3, 19, 36], Turkey (*n* = 2) [17, 31], China (*n* = 1) [18], India (n = 1) [21], Netherlands (n = 1) [24], and Tunisia (n = 1) [32]. The patients’ mean ages ranged from 22.8 years to 30.8 years. Surgery occurred mostly during the second trimester, except in the studies by Eom et al. [23] and Upadhyay et al. [33]. The negative appendectomy rate ranged from 0 to 42.9%. The complicated appendicitis rate ranged from 0 to 31.3%. Fetal losses were reported in 21 studies [3, 16–26, 31–39], and preterm deliveries were reported in 16 studies [16–26, 33, 35–38]. In addition, the birth weights were reported in eight studies [3, 16, 17, 19, 20, 23, 25, 35], and the Apgar scores were reported in six studies [3, 17, 19, 20, 25, 35].

**Table 1:** Baseline characteristics of the included studies

Reference	Year	Study design	Age (years)^a^	GA (weeks)^a^	No. of Women			Negative appendectomy (%)	Complicated appendicitis (%)	Outcomes
					Total	LA	OA			
Yoo et al. [16]	2016	Retrospective	30.8	20.2	80	24	56	NA	31.3	Fetal loss, preterm delivery, LOS, operative time, birth weight, wound infection, intraabdominal abscess
Karaman et al. [17]	2016	Retrospective	22.8	24.9	48	12	36	NA	NA	Fetal loss, preterm delivery, LOS, operative time, Apgar score, birth weight, wound infection, intraabdominal abscess
Cox et al. [30]	2016	Retrospective	27.9	NA	1335	894	441	NA	9.0	Operative time, wound infection, LOS, intraabdominal abscess
Cheng et al. [18]	2015	Retrospective	NA	NA	781	128	653	NA	15.5	Fetal loss, preterm delivery, LOS
Peled et al. [19]	2014	Retrospective	28.1	17.9	85	26	59	17.6	12.9	Fetal loss, preterm delivery, LOS, Apgar score, birth weight,
Kapan et al. [31]	2013	Retrospective	26.2	17.5	17	7	10	NA	NA	Fetal loss, LOS, operative time,
Chung et al. [20]	2013	Retrospective	30.6	16.6	61	22	39	9.8	11.5	Fetal loss, preterm delivery, LOS, operative time, Apgar score, birth weight, wound infection, intraabdominal abscess
Miloudi et al. [32]	2012	Retrospective	NA	NA	27	16	11	NA	14.8	Fetal loss
Khan et al. [21]	2012	Retrospective	22.8	17.3	118	52	66	NA	NA	Fetal loss, preterm delivery, LOS, operative time, wound infection, intraabdominal abscess
Jung et al. [22]	2012	Retrospective	27.9	15.4	25	4	21	0	NA	Fetal loss, preterm delivery, LOS, wound infection
Eom et al. [23]	2012	Retrospective	29.1	38.7	43	15	28	0	23.3	Fetal loss, preterm delivery, LOS, operative time, birth weight, intraabdominal abscess
De Bakker et al. [24]	2011	Retrospective	NA	NA	15	12	3	3.0	NA	Fetal loss, preterm delivery, LOS, operative time
Sadot et al. [25]	2010	Retrospective	29.5	19.7	65	48^b^	17^b^	24.1	12.3	Fetal loss, preterm delivery, LOS, operative time, Apgar score, birth weight, wound infection
Corneille et al. [26]	2010	Retrospective	25.6 (6.4)	15.9 (8.4)	49	9	40	NA	NA	Fetal loss, preterm delivery, LOS
Kirshtein et al. [3]	2009	Retrospective	28.4	13.9 (6.0)	42	23	19	42.9	19.0	Fetal loss, LOS, operative time, Apgar score, birth weight, wound infection
Upadhyay et al. [33]	2007	Prospective	27.2 (3.3)	32 (2.6)	6	4	2	NA	NA	Fetal loss, preterm delivery
McGory et al.	2007	Retrospective	27.2 (6.0)	NA	3133	454	2679	23.1	25.3	Fetal loss
Carver et al. [34]	2005	Retrospective	23.4 (5.8)	14 (5.4)	28	17	11	NA	NA	Fetal loss, preterm delivery, LOS, Apgar score, birth weight, wound infection
Lyass et al. [36]	2001	Prospective	28.5 (15.2)	20 (6.3)	22	11	11	31.8	0	Fetal loss, preterm delivery, LOS, operative time
Affleck et al. [37]	1999	Retrospective	NA	NA	37	19	18	NA	NA	Fetal loss, preterm delivery
Gurbuz et al. [38]	1997	Prospective	24.5 (1.5)	20.1 (9.0)	9	5	4	NA	NA	Fetal loss, preterm delivery, LOS, operative time
Curet et al. [39]	1996	Retrospective	NA	NA	11	4	7	NA	NA	Fetal loss

^a^Values are the means (standard deviations)

^b^Missing data, 41 vs 16

*NA* not available, *GA* gestational age, *LA* laparoscopic appendectomy, *OA* open appendectomy, *LOS* length of stay

### Pregnancy outcomes

**Fig. 2:**
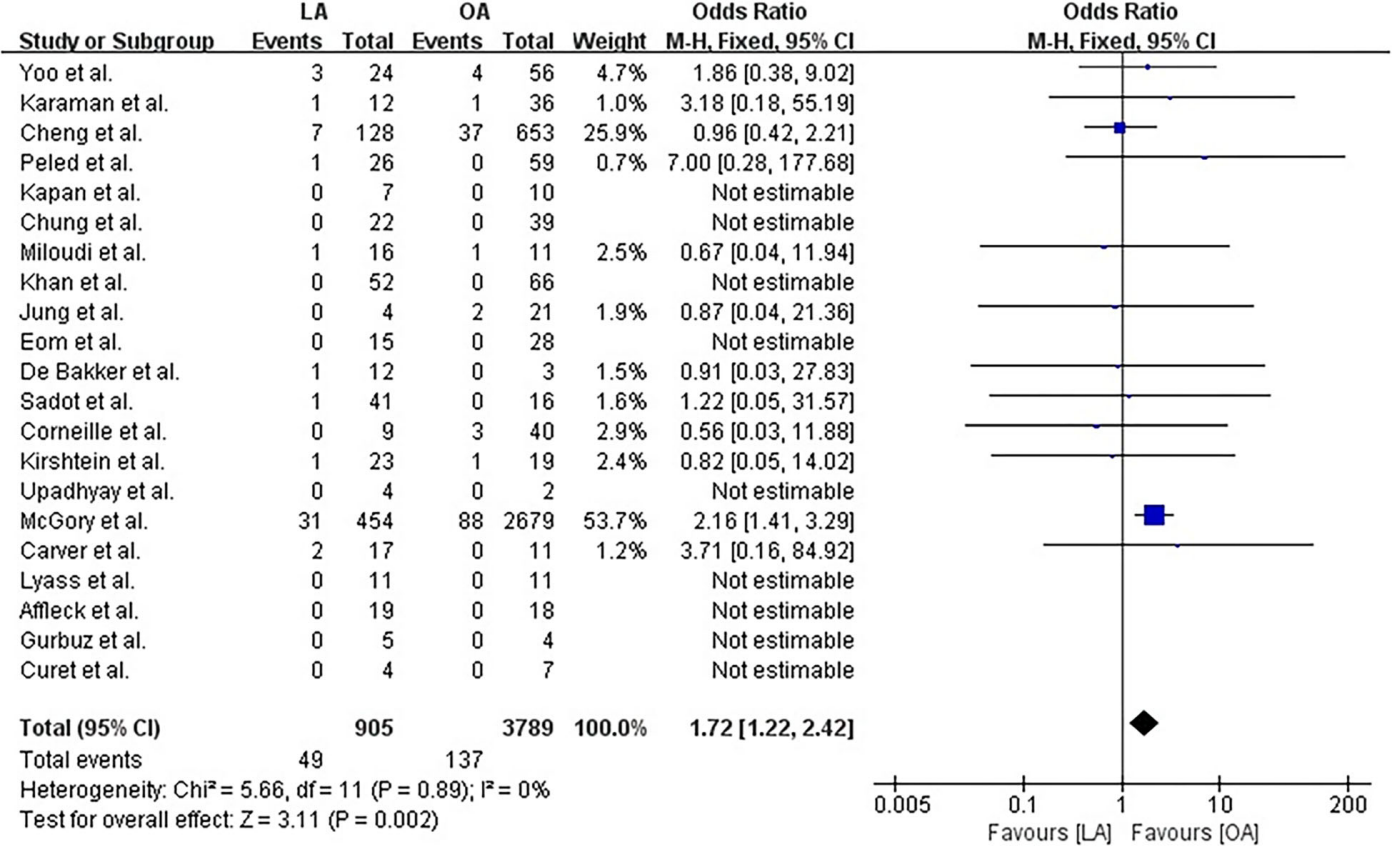
Forest plot comparing fetal loss after laparoscopic appendectomy versus open appendectomy. LA: laparoscopic appendectomy; OA: open appendectomy; CI: confidence interval

**Fig. 3:**
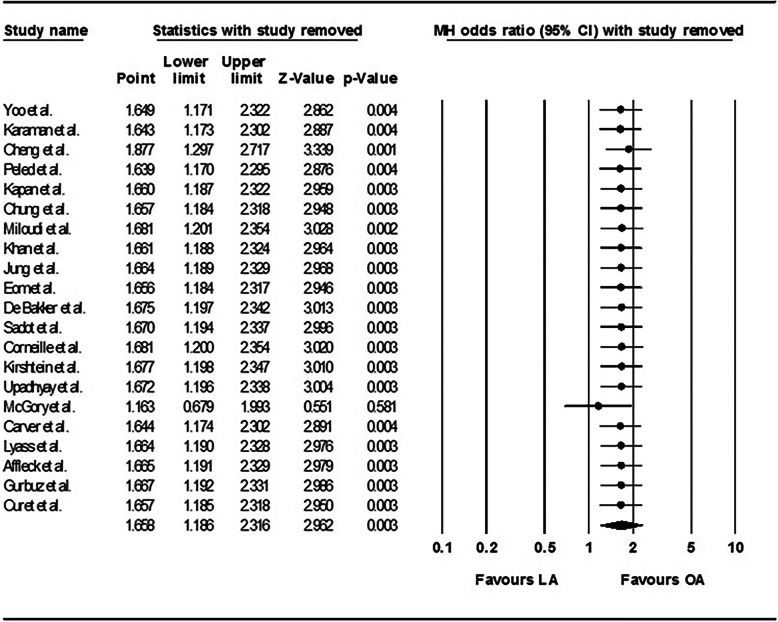
Sensitivity analysis that examined the influence of individual studies on the pooled estimates of fetal loss. LA: laparoscopic appendectomy; OA: open appendectomy; CI: confidence interval

The risk of fetal loss was significantly higher in the women who underwent LA compared with those who underwent OA, and the pooled OR was 1.72 (95% CI: 1.22–2.42) in the fixed effects model (*P* = 0.89; I^2^ = 0%) (Fig. 2). The one-study removed analysis showed that the study by McGory et al. [34] had a relatively strong influence on the results, and removing this study from the analysis showed that there was no significant difference between LA and OA with respect to the risk of fetal loss (OR 1.163, 95% CI: 0.68–1.99; *P* = 0.581) (Fig. 3).

**Fig. 4:**
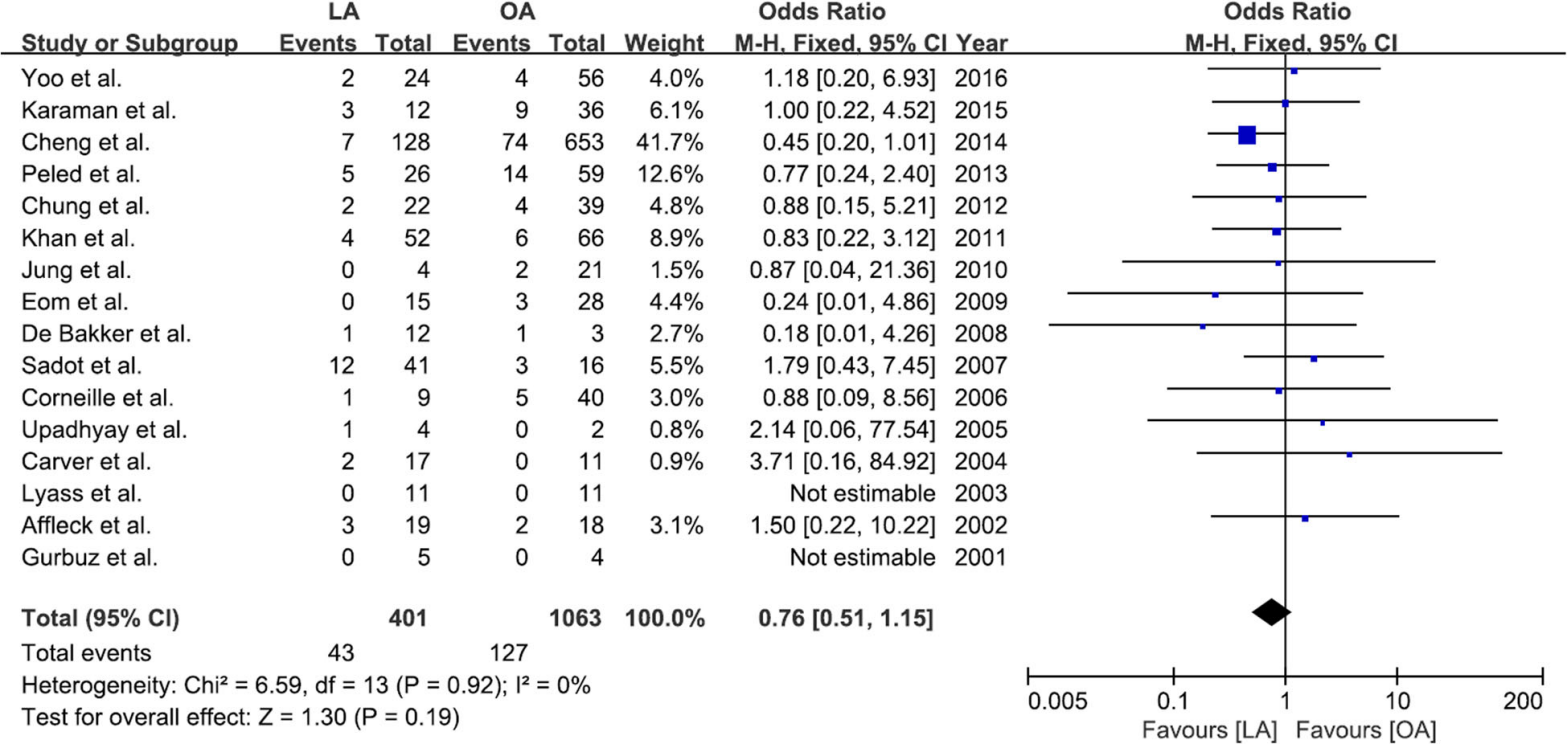
Forest plot comparing preterm delivery after laparoscopic appendectomy versus open appendectomy. LA: laparoscopic appendectomy; OA: open appendectomy; CI: confidence interval

**Fig. 5:**
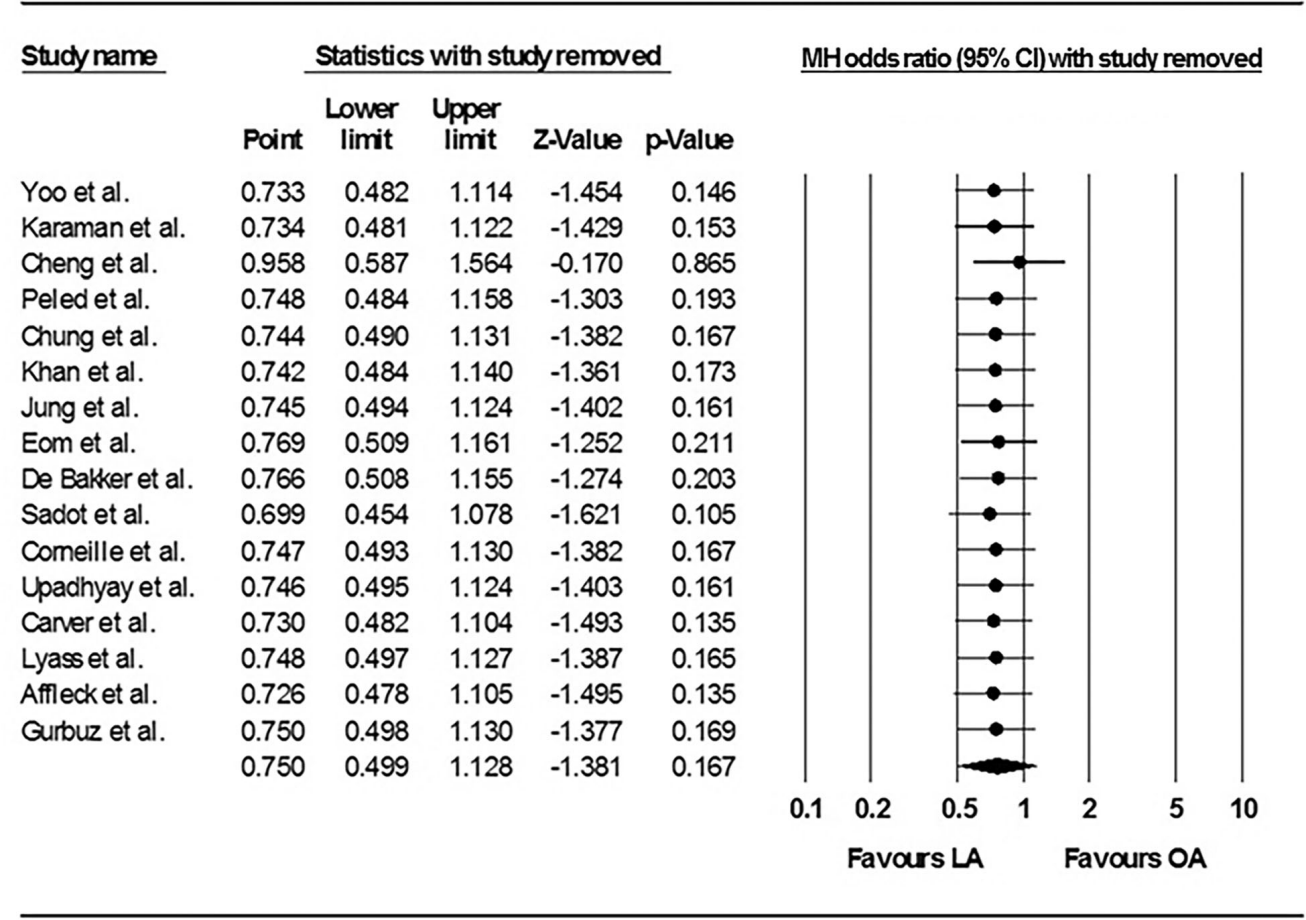
Sensitivity analysis that examined the influence of individual studies on the pooled estimates of preterm delivery. LA: laparoscopic appendectomy; OA: open appendectomy; CI: confidence interval

No significant difference was evident between LA and OA with respect to preterm delivery (OR 0.76, 95% CI: 0.51–1.15) in the fixed effects model (*P* = 0.92; I^2^ = 0%) (Fig. 4). Moreover, the sensitivity analysis did not show any changes in relation to preterm delivery after the exclusion of specific studies (Fig. 5).

The meta-analyses of the other pregnancy outcomes, for example, birth weight and the Apgar score, are shown in Table 2. The birth weight (*n* = 409) did not show a significant MD between LA and OA (MD 0.01, 95% CI: -0.09–0.10; *P* = 0.88) in the fixed effects model (*P* = 1.00; I^2^ = 0%). The LA and the OA groups were compared in relation to the Apgar scores in five studies (Apgar score at 1 min: *n* = 287; Apgar score at 5 min: *n* = 219). The data were heterogeneous (Apgar score at 1 min: *P* = 0.03; I^2^ = 66%; Apgar score at 5 min: *P* < 0.001; I^2^ = 84%), and the unstandardized MDs were 0.12 (95% CI: -0.18–0.08; *P* = 0.43) for the Apgar score at 1 min and − 0.02 (95% CI: -0.16–0.12; *P* = 0.76) for the Apgar score at 5 min, indicating that there were no significant differences between the groups with respect to the Apgar scores.

**Table 2:** Summary of the meta-analyses of the pregnancy and surgical outcomes from laparoscopic appendectomy and open appendectomy

	Number of Studies	Pooled number of patients (LA/OA)	Test for Heterogeneity (*P* value, I^2^)	Model	Pooled outcome	*P* value*
Birth Weight (gram)	7	172/237	1.00, 0%	Fixed	MD: 0.01(−0.09–0.10)	0.88
Apgar (1 min)	5	125/162	0.03, 66%	Random	MD: 0.12(− 0.18–0.08)	0.43
Apgar (5 min)	4	113/106	< 0.001, 84%	Random	MD: − 0.02(− 0.16–0.12)	0.76
Wound Infection (n)	7	1096/706	0.15, 36%	Fixed	OR: 0.40 (0.21–0.76)	0.005
Operative Time (min)	8	1082/684	< 0.001, 80%	Random	MD: 2.23(−3.20–7.65)	0.42
Hospital Stay (days)	10	1106/756	< 0.001, 86%	Random	MD: −1.01(−1.61– −0.41)	0.001
Intra-abdominal Abscess (n)	7	1090/702	0.55, 0%	Fixed	OR: 0.79 (0.33–1.85)	0.58

*P value for pooled result

*OR* odds ratio, *MD* mean difference

### Surgical outcomes

Data describing the wound infection and intra-abdominal abscess rates, the operative times, and the LOS were pooled across the studies (Table 2). Nine of the studies reported the wound infection rates [3, 16, 17, 20–22, 25, 30, 35], and the pooled results showed that there was a significantly lower risk of wound infection in the LA group compared with that in the OA group (OR 0.40, 95% CI: 0.21–0.76; *P* = 0.005). Seventeen of the studies reported the LOS [3, 16–26, 30, 31, 35, 36, 38], and, of these, 12 reported the means and standard deviations [3, 16, 17, 19–22, 25, 26, 30, 31, 35]. The five remaining studies did not report the standard deviations [18, 23, 24, 36, 38], and two studies reported the medians and interquartile ranges [18, 24]; therefore, they could not be used in the meta-analysis. Ten of the studies defined the LOS as the “length of the hospital stay” and two studies [3, 26] defined the LOS as the “postoperative LOS”. Only the “length of the hospital stay” was used in the meta-analysis. The mean LOS was significantly shorter in the LA group compared with that in the OA group (MD -1.01, 95% CI: -1.61–-0.41; *P* = 0.001), but the analysis showed heterogeneity (*P* < 0.001; I^2^ = 86%).

Twelve of the studies reported the operative times [3, 16, 17, 20, 21, 23–25, 30, 31, 36, 38], and, of these, eight reported the means and standard deviations [3, 16, 17, 20, 21, 25, 30, 31]. The four remaining studies did not report the standard deviations [23, 24, 36, 38], and one study [24] reported the median and the interquartile range; therefore, these studies could not be used in the meta-analysis.There were no significant differences between the LA and the OA groups with respect to the operative time (MD 2.23, 95% CI: -3.20–7.65; *P* = 0.42) or the intra-abdominal abscess rate (OR 0.79, 95% CI: 0.33–1.85; *P* = 0.58) [3, 16, 17, 20, 21, 23, 25, 30].

### Meta-regression analysis

**Fig. 6:**
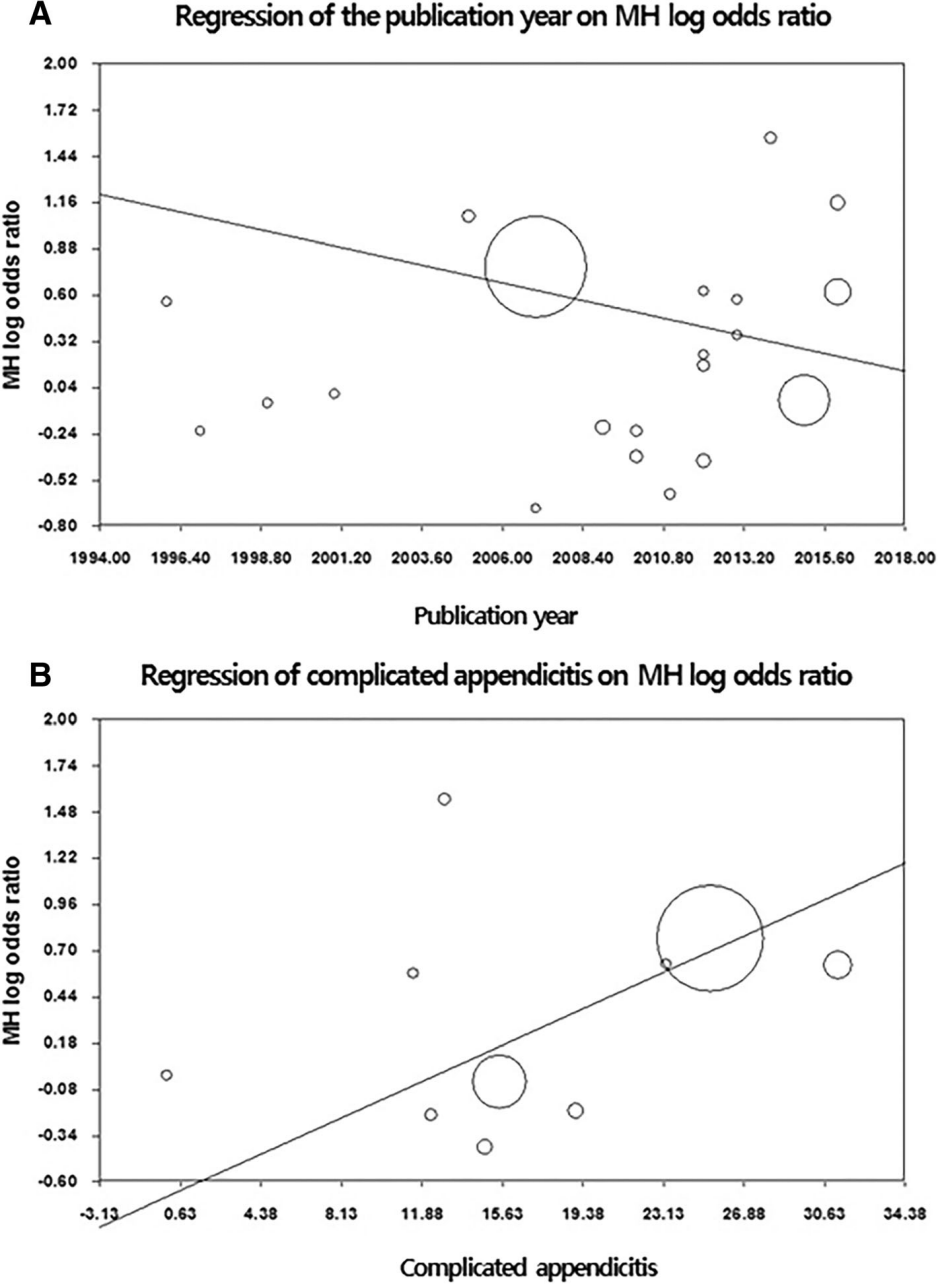
Scatter plots of the meta-regression analyses of the effects of the publication year (**a**) and complicated appendicitis (**b**) on the odds ratios for fetal loss

Meta-regression analyses were conducted to evaluate whether the year in which the study was published, the presence of complicated appendicitis, the gestational age at surgery, the trimester, or the presence of a negative appendectomy influenced the meta-analysis outcomes, for example, fetal loss and preterm delivery. None of the meta-regression analyses were statistically significant (Table 3). The meta-regression analyses showed trends towards a decreasing OR for fetal loss in association with a more recent publication year and an increasing OR for fetal loss in association with a higher complicated appendicitis rate (Fig. 6).

**Table 3:** Meta-regression analyses of the effects of each covariate on fetal loss and preterm delivery

	Fetal loss				Preterm delivery			
Covariates	Number of studies	Point estimate	95% CI	*P* value	Number of studies	Point estimate	95% CI	*P* value
Publication year	21	−0.045	(−0.130–0.040)	0.304	16	−0.055	(−0.153–0.043)	0.271
Mean age	16	−0.008	(−0.322–0.305)	0.957	13	0.013	(−0.188–0.214)	0.902
Mean GA	15	0.007	(−0.140–0.153)	0.93	13	−0.028	(−0.142–0.086)	0.633
Proportion of 1st	9	−0.02	(−0.102–0.062)	0.635	8	0.001	(−0.050–0.052)	0.97
Proportion of 2nd	9	0.003	(−0.080–0.060)	0.952	8	0.004	(−0.040–0.048)	0.849
Proportion of 3rd	9	0.012	(−0.054–0.347)	0.729	8	−0.005	(−0.042–0.033)	0.8
Proportion of CA	10	0.055	(−0.015–0.124)	0.123	7	−0.002	(−0.095–0.091)	0.969
Proportion of NA	13	0.009	(−0.057–0.281)	0.778	9	0.032	(−0.062–0.126)	0.499

*CI* confidence interval; *GA* gestational age, *CA* complicated appendicitis, *NA* negative appendicitis

### Publication bias

**Fig. 7:**
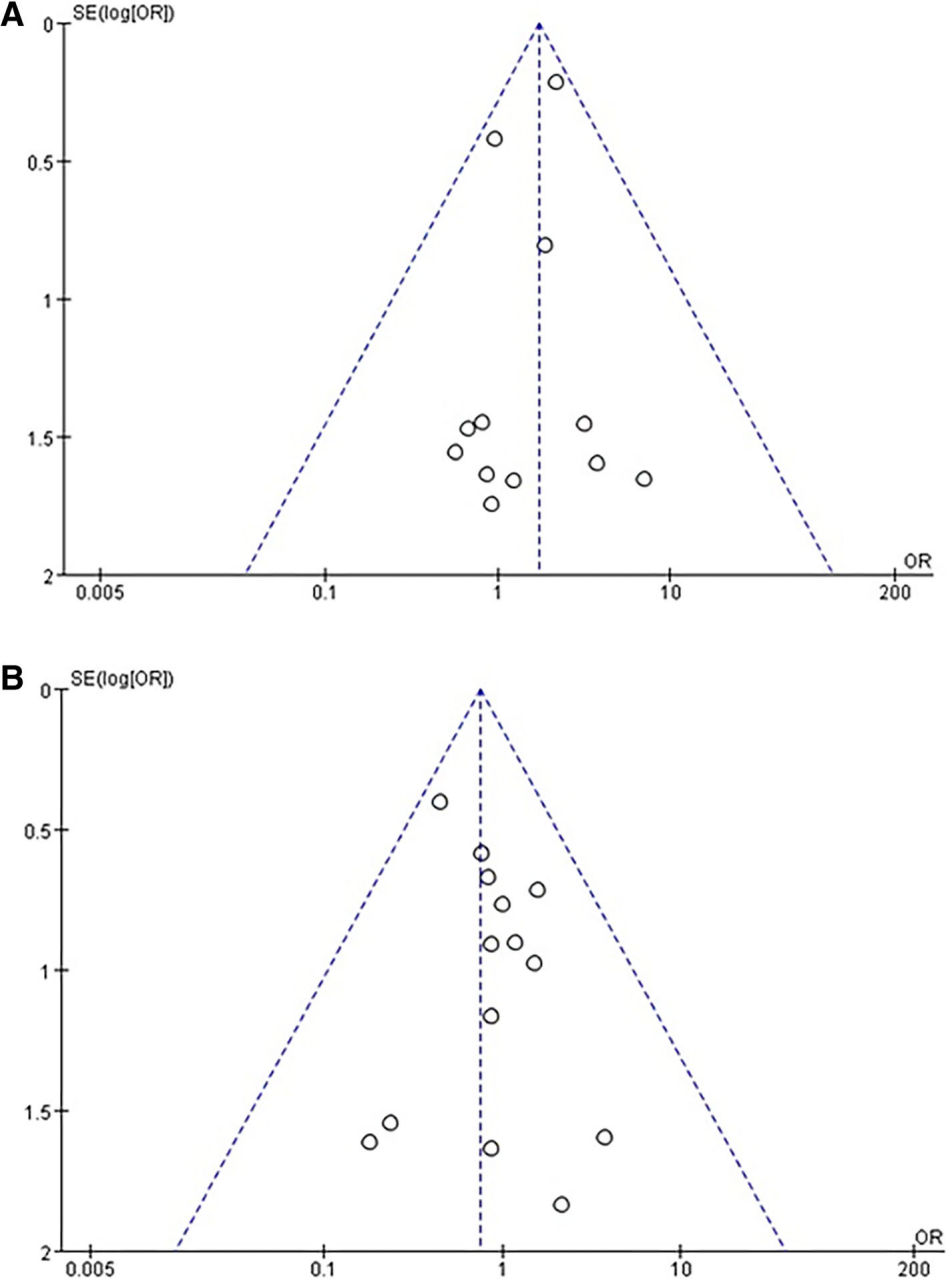
Funnel plots of the studies that described fetal loss (**a**) and preterm delivery (**b**)

The funnel plots for fetal loss and preterm delivery are presented in Fig. 7. The contours are almost symmetrical, indicating that there was no evidence of publication bias.

## Discussion

The findings from this systematic review and meta-analysis showed that it is not reasonable to conclude that LA during pregnancy might be associated with a greater risk of fetal loss. Indeed, no significant differences were observed between the LA and OA groups with respect to preterm delivery, the birth weight, the Apgar scores, the operative time, or intra-abdominal abscess formation after surgery. The results from this meta-analysis also showed that compared with OA, LA might be associated with a lower risk of wound infection and a shorter LOS.

According to the current SAGES guidelines, LA may be performed safely in pregnant patients who have a suspicion of appendicitis [28]. The present authors also prefer LA over OA for pregnant patients with presumed appendicitis because of its effectiveness in access to the appendix, visualization, and reduce surgical complications. Although our data are not yet enough to evaluate pregnancy outcomes in pregnant women who underwent LA, there was no complication such as fetal loss after LA. Furthermore, the publications from recent studies have reported that LA can be performed safely during any pregnancy trimester [16, 17, 20, 33]. However, both a systematic review [5] and a meta-analysis [4] concluded that there was low grade evidence to suggest that LA in pregnant women might be associated with a greater risk of fetal loss. Thus, the safety of LA during pregnancy remains controversial.

The findings from the current updated meta-analysis showed that fetal loss was significantly higher in pregnant women who underwent LA compared with those who underwent OA. However, the sensitivity analysis revealed that this finding was greatly influenced by the study undertaken by McGory et al. [34] that had the largest sample size among the pooled studies. Although there was no publication bias and heterogeneity was not evident in this analysis, meta-regression analyses were performed to determine whether fetal loss was associated with the publication year, complicated appendicitis, the gestational age, the trimester, or negative appendectomy. None of the meta-regression analyses were statistically significant. However, the meta-regression analyses showed trends towards a decreasing OR for fetal loss in association with a more recent publication year and an increasing OR for fetal loss in association with a higher complicated appendicitis rate. These results are supported by those from more recent studies [3, 16–19, 25] that showed that, with the exception of two studies [34, 35], LA appeared to be a safe, feasible, and efficacious approach during pregnancy. Moreover, a previous study’s findings demonstrated that fetal outcomes are more likely to be adversely affected by the type of infection and misdiagnosed disease rather than by the laparoscopic approach itself [24]. Therefore, it is not reasonable to conclude that LA during pregnancy might be associated with a greater risk of fetal loss.

The effect of pneumoperitoneum is a major consideration in relation to laparoscopic surgery during pregnancy. Increasing the intra-abdominal pressure to induce pneumoperitoneum can reduce the venous return and cardiac output [40], resulting in maternal hypotension and hypoxia [41]. In addition, fetal acidosis may occur as a consequence of carbon dioxide pneumoperitoneum [42]. However, the findings from a previous study undertaken on an animal pregnancy model showed that the fetus was not adversely affected when the pneumoperitoneal pressure was elevated to 10–12 mmHg for less than 30 min [43]. Furthermore, the SAGES guidelines recommend insufflation pressures of 10–15 mmHg for pregnant patients [28]. Therefore, there was insufficient evidence to determine whether the risk of fetal loss was greater in association with LA or OA.

Although the precise cause of preterm delivery after surgery during pregnancy remains unclear, the findings from previous studies have indicated that preterm delivery was associated with uterine irritability during the operations [44, 45]. While the results from this updated meta-analysis showed that there was no significant difference between LA and OA with respect to preterm delivery, a trend towards an increasing risk of preterm delivery was evident in those who underwent OA compared with those who underwent LA, which differed from the findings from a previous meta-analysis [4]. Furthermore, the meta-regression analysis showed a trend towards a decreasing OR for preterm delivery in association with a more recent publication year, but the meta-analyses did not determine any significant differences between LA and OA with respect to the other pregnancy outcomes, for example, the birth weight and the Apgar scores, which concurs with the results from a previous meta-analysis [4].

Interestingly, the pooled results from this updated meta-analysis that were related to the primary outcomes, namely, fetal loss and preterm delivery, showed opposing trends in the two groups. These results could be attributed to a single study even though the two outcomes may be caused by different factors, for example, pneumoperitoneum and uterine irritability during the operation. If fetal loss and preterm delivery are caused by pneumoperitoneum and uterine irritability, respectively, during the operation, it is very interesting that the pooled results showed opposing trends in the two groups.

Postoperative complications are usually considered to evaluate the safety of surgical procedures. The results from this meta-analysis showed that the risk of wound infection was significantly lower in the LA group compared with that in the OA group. In contrast, the findings from a previous meta-analysis suggested that there was no significant difference between LA and OA during pregnancy with respect to wound infection [4]. However, the findings from a recently published meta-analysis showed that the rate of wound infection in the general population was significantly lower in the LA group compared with that in the OA group [46]. Moreover, the results from the current meta-analysis showed a tendency, albeit one that was not statistically significant, to favor LA because of the lower risk of intra-abdominal abscesses, which concurs with the results from the previous meta-analysis that used data from the general population [46].

The results from the present meta-analysis revealed that the mean LOS was significantly shorter in the LA group compared with that in the OA group, a finding that concurs with those reported from previous meta-analyses that used data from pregnant women and the general population [4, 46]; hence, this can be considered a benefit of LA. The operative time was longer in the LA group compared with that in the OA group, but the difference between the groups was not statistically significant. This result concurs with that from a meta-analysis that used data from pregnant women [4]. Moreover, the findings from a recent study of the general population suggested that LA took significantly longer to complete than OA [46]. Thus, LA shows a trend towards longer operative times compared with OA in pregnant women.

We updated the meta-analysis that was conducted during a previous related study. This meta-analysis evaluated the effects of LA and OA on pregnancy and surgical outcomes. One of the study’s strengths was related to the sensitivity and meta-regression analyses of the pregnancy outcomes that were performed to corroborate the findings from the meta-analysis. Another strength of this study was the rigorous literature search. Despite these strengths, there are some limitations to the present study. First, there were no randomized controlled trials among the studies analyzed. Second, the studies analyzed tended to involve small study populations. Third, the outcome assessments differed and different definitions for each variable existed among the studies.

## Conclusions

In summary, the sensitivity analysis of the studies that comprised this meta-analysis showed that one study by McGory et al. [34] had a disproportionately high influence on the findings from this meta-analysis, and that when this study [34] was removed from the analysis, no significant difference was evident between LA and OA in relation to the risk of fetal loss. Thus, the findings from this systematic review and updated meta-analysis show that it is not reasonable to accept without question the conclusions from a previous systematic review and meta-analysis that indicated that LA in pregnant women might be associated with a greater risk of fetal loss. Furthermore, there was no significant difference between the LA and OA groups with respect to preterm delivery. Compared with OA, LA was associated with lower wound infection rates and shorter LOS. Based on our results and recent literatures, we suggest that LA shows non-inferior safety with respect to pregnancy outcomes but superior with regard to surgical outcomes compared with OA in pregnant women with suspected appendicitis. Although it is difficult to conduct randomized trials on pregnant women, large scale and well-designed trials are needed to clarify the present findings. This systematic review and updated meta-analysis will help guide surgeons in their decision making in relation to treatment options for appendicitis during pregnancy.

## Abbreviations

CI: Confidence interval
GA: Gestational age
LA: Laparoscopic appendectomy
LOS: Length of stay
MD: Mean difference
OA: Open appendectomy
OR: Odds ratio
